# Marktvaccinaties in Rotterdam: Een voorbeeld van een campagne gestoeld op het equity-principe

**DOI:** 10.1007/s12508-022-00355-w

**Published:** 2022-07-11

**Authors:** Inge Merkelbach, Shakib Sana, Tessa Magnée, Paul Kocken, Robin Peeters, Semiha Denktaș

**Affiliations:** 1 Erasmus School of Social & Behavioural Sciences, Erasmus Universiteit Rotterdam, Rotterdam, Nederland; 2 Inwendige geneeskunde, Erasmus Medisch Centrum, Rotterdam, Nederland

**Keywords:** equity, equality, vaccinatie, lage SES, outreach, Equity, Equality, Vaccination, Low SES, Outreach

## Abstract

De COVID-19-vaccinatiegraad in Nederland is niet gelijk verdeeld: in sommige wijken blijft de vaccinatiegraad achter. Dat leidt tot individuele gezondheidsrisico’s en belasting van de zorgketen. Een verklaring hiervoor is dat een gelijke aanpak (*equality*-aanpak) niet altijd tot gelijke uitkomsten leidt, terwijl er op landelijk niveau wel gekozen is voor een algemene voorlichtingscampagne. Wij bepleiten daarom het inzetten van de *equity*-aanpak, zoals door de WHO gedefinieerd, gericht op het behalen van gelijke resultaten door in te spelen op de specifieke behoeften van kwetsbare groepen. Als voorbeeld beschrijven we een Rotterdamse interventie, waarbij Rotterdamse (huis)artsen het initiatief namen informatie over vaccins en vaccinatie aan te bieden op de drukbezochte markten van Rotterdam. Aan de hand van interviews met betrokken medische vrijwilligers schetsen wij daarnaast enkele randvoorwaarden voor het succesvol inzetten van een dergelijke op outreach gebaseerde aanpak en doen we enkele praktische aanbevelingen.

## Abstract (en)

The COVID-19 vaccination rate is not evenly distributed across the Netherlands. In some neighborhoods, the vaccination rate is relatively low, causing individual health risks and a burden to the health care system. An explanation is the use of a national, general information campaign, while using a similar approach (equality approach) does not always lead to similar results. Therefore, we advocate the use of an equity approach instead, as defined by the WHO, which aims at achieving equal results by taking the specific needs of certain vulnerable groups into account. As an example, we describe an intervention that took place in Rotterdam, in which medical doctors, among which GPs, took the initiative to offer information about vaccinations and vaccination on the popular weekly markets of Rotterdam. Using interviews with medical volunteers who contributed to these market vaccinations, we outline the preconditions for successfully implementing such an outreach-based approach, and we make a number of practical recommendations.

## Rotterdamse resultaatgerichte marktvaccinaties – een equity-benadering

De COVID-19-vaccinatiegraad is in Nederland ongelijk verdeeld. De vaccinatiegraad blijft achter in de grote steden. Vooral in wijken waar het gemiddelde opleidings- en inkomensniveau laag ligt, wonen relatief veel niet of onvolledig gevaccineerden [1, 2]. Deze lage vaccinatiegraad leidt tot grote risico’s voor de individuele gezondheid van wijkbewoners en tot extra druk op de zorgketen. Dit wordt versterkt door het feit dat chronische ziekten, zoals diabetes [3], die het risico op een gecompliceerd beloop van COVID-19 verhogen, in deze wijken relatief veel voorkomen [4].

Vaccinatie is en blijft een vrije keuze. De vraag is echter of mensen in achterstandswijken al dan niet actief de keuze maken zich *niet* te laten vaccineren, en hoe dit kan worden verklaard. Hebben zij wel toegang tot de juiste informatie en zijn er misschien praktische barrières die een vaccinatie in de weg staan? Wij zijn van mening dat het mogelijk en wenselijk is beleid te voeren dat erop gericht is de oorzaken van de lage vaccinatiegraad weg te nemen, naar behoefte communicatie te bieden en een gelijkere vaccinatiegraad te realiseren.

## Waar komt de lage vaccinatiegraad in kwetsbare wijken vandaan?

Het achterblijven van de vaccinatiegraad in achterstandswijken is niet door één oorzaak te verklaren. Er is sprake van een complexe opeenstapeling van factoren, zoals onder andere beperkte toegang tot juiste informatie, vertrouwen in de werking en veiligheid van het vaccin, minder vertrouwen in de overheid en ook beperkte vervoersopties naar vaccinatielocaties. Deze factoren zorgen onder inwoners van kwetsbare wijken voor een hogere drempel om zich te laten vaccineren, en daardoor een verminderde vaccinatiebereidheid [5, 6].

**Figure 1:**
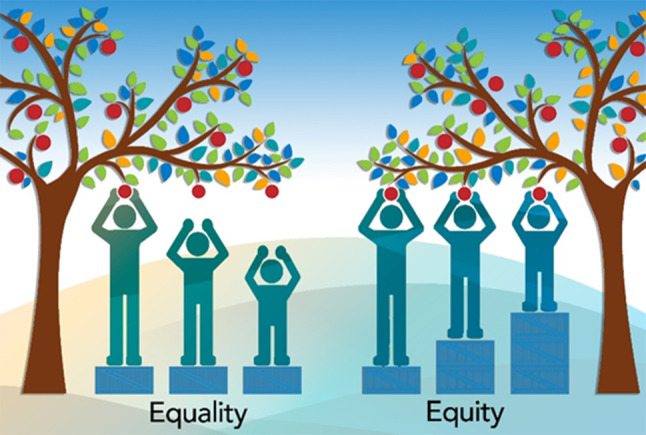

Het ministerie van VWS lanceerde een grootschalige, algemene vaccinatie- en voorlichtingscampagne. Iedereen kreeg dezelfde informatie- en vaccinatiemogelijkheid aangeboden. In dit verband is het goed om kennis te nemen van het onderscheid dat de Wereldgezondheidsorganisatie (WHO) maakt tussen *equality* en *equity* (zie fig. 1). Bij beleid dat gestoeld is op het equality-principe gaan we ervan uit dat iedereen baat heeft bij dezelfde informatie, dezelfde ondersteuning en hetzelfde zorgaanbod. Bij beleid dat gestoeld is op het equity-principe erkennen we dat verschillende individuen en groepen andere informatie, ondersteuning en zorg nodig hebben om vergelijkbare resultaten te bereiken.

De onevenredig grote last van COVID-19-morbiditeit en -mortaliteit bij kwetsbare groepen als gevolg van de ongelijke vaccinatiegraad in Rotterdamse wijken was voor medische professionals aanleiding om een op het equity-principe gebaseerde communicatie- en vaccinatiecampagne te starten [1].

In dit artikel beschrijven we deze campagne en doen we daarnaast verslag van de interviews die we afnamen bij medische professionals die aan de campagne meewerkten. We wilden weten wat hun motivatie was voor deelname en hoe ze de kwaliteit van deze campagne beoordeelden. Daarnaast spraken we met medewerkers van de GGD. Aan de hand van hun antwoorden geven we een beeld van de campagne gezien vanuit de betrokken professionals, schetsen we een aantal randvoorwaarden voor een succesvolle fijnmazige campagne en doen we aanbevelingen voor toekomstige campagnes.

## De Rotterdamse marktvaccinaties

In juni 2021 startten medische professionals in Rotterdam een communicatie- en vaccinatiecampagne gestoeld op het equity-principe. Veel activiteiten in de infectiebestrijding of gezondheidsbevordering van GGD’en gaan uit van dit principe. Vanwege de volksgezondheidsdreiging kwam een doelgroepgerichte benadering bij COVID-19-vaccinaties echter laat opgang. Hoewel de marktvaccinaties pasten binnen de visie van de GGD en door de GGD gesteund werden, lagen het initiatief en het agenderen van de urgentie van deze aanpak in eerste instantie geheel bij de medische professionals. Vanwege personeelstekort binnen de GGD lagen ook de medische eindverantwoordelijkheid en het geven van informatie op locaties tijdens de start van de campagne bij de artsen. Gedurende de campagne werkten artsen en GGD’en steeds meer samen om deze tot een succes te maken.

De campagne, die als snel de naam ‘marktvaccinaties’ kreeg, werd aangeboden op de drukbezochte weekmarkten in de Rotterdamse wijken Noord, Delfshaven en Feijenoord. Informatie werd gegeven naar gelang de behoefte van de marktbezoekers en de vaccinatie kon ter plekke, zonder afspraak, worden gezet. Daarnaast waren mensen vrij in het kiezen van het type vaccin dat ze wensten te ontvangen. De campagne werd uitgevoerd door veertig tot zestig vrijwilligers met een medische achtergrond (bijvoorbeeld specialisten, huisartsen, medische studenten en verpleegkundigen), die zeven zaterdagen in deze wijken actief waren. Vrijwilligers hadden verschillende culturele achtergronden – naast vrijwilligers met een Nederlandse achtergrond waren er onder andere ook vrijwilligers met een Turkse, Marokkaanse of Surinaamse achtergrond aanwezig. De vrijwilligers voerden verschillende taken uit, zoals voorlichten, helpen met het invullen van gezondheidsvragenlijsten, patiëntregistratie, vaccineren en monitoren na vaccinatie. In totaal werden er in logistieke samenwerking met GGD Rotterdam op deze manier 2.200 vaccinaties gezet.

### Het onderzoek

We onderzochten de mening van zestien betrokken medische vrijwilligers door hen te bevragen. We selecteerden vrijwilligers met diverse medische achtergronden (huisartsen, specialisten, verpleegkundigen en studenten), om een zo volledig en representatief mogelijk beeld te krijgen. Vrijwilligers werden bevraagd aan de hand van een semigestructureerd interview over hun visie op de aanpak. Het interview werd vormgegeven aan de hand van de zelfdeterminatietheorie en het RE-AIM-model [7, 8]. Het interview vond digitaal plaats, en werd opgenomen en getranscribeerd.

Om bevindingen in de juiste context te plaatsen interviewden we vervolgens een locatie- en een programmamanager van de lokale GGD over hun visie op de aanpak. Beiden waren nauw betrokken bij de marktvaccinaties.

Voor dit onderzoek is toestemming verkregen van de Ethische Toetsingscommissie van de Erasmus School of Social and Behavioural Sciences (ESSB), Erasmus Universiteit Rotterdam.

## Barrières en oplossingen

In de afgenomen interviews noemden de medische professionals een aantal barrières die relatief eenvoudig met de marktvaccinatiescampagne konden worden geadresseerd (zie tab. 1).

**Table 1:** 

barrière	oplossing
geen vervoer naar moeilijk bereikbare vaccinatielocaties Aios: ‘Wat ik door mijn inzet tijdens de marktvaccinaties heb geleerd? Dat het uiteindelijk toch vaak neerkomt op praktische bezwaren of motieven: niet op een priklocatie kunnen komen, op vakantie willen gaan. Mensen hebben veel simpelere redenen om wel of niet voor een vaccinatie te kiezen dan de ideologische overtuigingen waarover je vaak hoort.’	vaccinaties worden gezet op nabije en vaak bezochte locaties (drie Rotterdamse markten)
moeite met het maken van een (online) afspraak Huisarts: ‘Wie bereikt zijn met de marktvaccinaties? Met name mensen die het lastig vinden om naar de GGD-prikstraat te gaan. Die het een drempel vinden om te moeten bellen of vragen te moeten beantwoorden, of online niet vaardig zijn.’	vaccinaties op de markt worden ter plekke gezet
gebrek aan juiste en betrouwbare informatie Huisarts: ‘Ik schrok soms van wat er aan desinformatie bij mensen aanwezig was.’	medische professionals geven voorlichting op de markt
angst en onzekerheid over de betrouwbaarheid van de informatie Huisarts: ‘Het is nu een discussie die je niet meer gaat winnen met argumenten, maar met vertrouwen.’	inzet van medische professionals, zoals de (huis)arts
gebrek aan informatie in de eigen taal Praktijkondersteuner: ‘Talen beheersen van de doelgroep die moeilijk bereikbaar is en die het nieuws niet zo goed kan volgen, is noodzakelijk. Verder goed kunnen informeren over vaccinaties, vakinhoudelijk goed op de hoogte zijn. Gelukkig waren er ook artsen die heel specifieke vragen konden beantwoorden, bijvoorbeeld een longarts.’	flyers zijn beschikbaar in verschillende talen en het team van vrijwilligers bestaat uit professionals met verschillende culturele achtergronden
niet stilstaan bij/bezig zijn met (het belang van) vaccinatie omdat er urgentere problemen spelen in het dagelijks leven (zoals armoede) Arts in opleiding tot internist: ‘We staan dichter bij de mensen. In plaats van ze naar ons laten toe komen, komen we naar hen toe. Er is een toereikende hand.’	informatie- en vaccinatielocaties zijn duidelijk zichtbaar in het straatbeeld, waardoor het makkelijker gemaakt wordt het gedrag te vertonen, ondanks dagelijkse problematiek

Medewerkers van de GGD bevestigden dat de marktvaccinaties voor bewoners van deze wijken een groot aantal drempels tot vaccinatie wegnamen, waarbij de mogelijkheid tot vragen stellen (in de eigen taal) als cruciaal werd gezien.

## De randvoorwaarden voor de marktvaccinatiescampagne

Om de randvoorwaarden voor deze campagne in kaart te brengen vroegen we medische vrijwilligers naar hun motivatie voor deelname aan de hand van de *zelfdeterminatietheorie *[7]. Deze theorie stelt dat voor intrinsieke motivatie een gevoel van verbondenheid, competentie en autonomie nodig zijn. Daarnaast vroegen we hoe ze de kwaliteit van de aanpak beoordelen aan de hand van het RE-AIM-model, een raamwerk voor het evalueren van interventies aan de hand van vijf componenten [8].

### Intrinsiek gemotiveerde vrijwilligers

Intrinsiek gemotiveerde vrijwilligers bleken essentieel voor het succes van de huidige aanpak. Ze namen deel omdat ze geloofden in het belang van de campagne en omdat de aanpak aansloot bij hun persoonlijke overtuigingen en doelen. Wanneer vrijwilligers terugkeken op de vaccinatieaanpak in zijn algemeenheid waren ze zeer positief, vooral vanwege het grote belang dat ze aan vaccinatie hechtten. ‘Elke prik telt’, was een vaak gehoorde uitspraak. Deze motivatie kwam ook tot uiting in het feit dat alle vrijwilligers aangaven opnieuw mee te willen doen wanneer er weer een dergelijke aanpak opgestart zou worden.

### Verbondenheid

Professionals namen over het algemeen deel omdat ze zich verbonden voelden met anderen: ze voelden bijvoorbeeld een verantwoordelijkheid jegens de maatschappij als geheel, en jegens de inwoners van achterstandswijken in het bijzonder. Dit was vooral het geval voor medische vrijwilligers die ook in het dagelijks werk in contact kwamen met bewoners van achterstandswijken. Zij noemden vaak dat ze al eerder hadden ervaren dat voor deze groepen een unieke, meer persoonlijke communicatieaanpak noodzakelijk was. Professionals die minder bekend waren met deze doelgroep, waren over het algemeen ook voor de start van de campagne al overtuigd van het nut van een dergelijke aanpak, maar gaven in sommige gevallen aan dat ze de omvang van de misvattingen rond vaccinatie hadden onderschat, en dat deelname aan deze aanpak ook daarom leerzaam was geweest.

Waardering van collega’s was geen reden om deel te nemen.

### Competentie

Vrijwel alle professionals gaven aan dat ze tijdens de vaccinatieaanpak zowel hun medische als sociale vaardigheden en kennis konden inzetten en benutten. Ook bekendheid met diverse culturen en talen werd door sommige vrijwilligers als belangrijk gezien en was soms essentieel voor het leggen van contact. Vrijwilligers die deze kennis ontbeerden, gaven aan afhankelijk te zijn van andere vrijwilligers (met deze kennis) voor het leggen van het eerste contact.

### Autonomie

Alle vrijwilligers gaven aan dat deelname hun persoonlijke keuze was geweest en niet werd opgedragen door de organisatie waar ze werkten.

## Interventie-elementen

### Bereik

Vrijwilligers gaven aan dat ze dachten dat de aanpak goed aansloot bij de behoeften van inwoners van achterstandswijken (zie ook tab. 1), vooral voor mensen met een immigratieachtergrond, taalproblemen, een beperkte toegang tot (openbaar) vervoer en een beperkte gezondheidsgeletterdheid, en voor mensen die twijfelden over vaccinatie door gebrek aan betrouwbare informatie. Vrijwilligers achtten zowel het aanreiken van informatie door medische professionals uit de wijk, als het geven van informatie in de eigen taal van essentieel belang. Groepen die niet bereikt werden waren volgens professionals jongeren en dak- en thuislozen, omdat zij de markt zelden bezochten. Goede aansluiting bij de behoeften van de doelgroep bleek essentieel.

### Effectiviteit

Over het algemeen beoordeelden de vrijwilligers de aanpak als effectief en schatten ze in dat veel mensen die op de markt voor vaccinatie kozen, anders niet gevaccineerd zouden worden.

### Beslissen tot deelname

Hoewel ze zelf tot deelname besloten, gaven sommige vrijwilligers aan dat werkgevers deelname afraadden of ontmoedigden vanwege onduidelijkheid rond de eindverantwoordelijkheid voor deze aanpak. Hoewel het initiatief bij de curatiesector (artsen) lag, vonden veel vrijwilligers deze aanpak eerder bij de preventiesector horen (bijvoorbeeld de GGD). Hierdoor ontstond soms onduidelijkheid over wie de aanpak coördineerde, wat voor enkelen onprettig was. Steun vanuit de werkgever en duidelijkheid rond coördinatie en verantwoordelijkheid zouden deze aanpak kunnen versterken.

### Implementatie

De uitvoering van de vaccinaties leidde onvermijdelijk tot onverwachte situaties of knelpunten. Ter plekke werden ad-hocbeslissingen genomen. Dit paste volgens de vrijwilligers bij het karakter van de aanpak. Er was sprake van *gecontroleerde improvisatie,* waarbij pragmatiek voorop stond. De benodigde (tijds)investering werd als hoog maar proportioneel beoordeeld, en het belang rechtvaardigde volgens de vrijwilligers zowel de financiële kosten als de vrijwillige inzet.

### Continuering of behoud

Vrijwilligers achtten toegankelijke een-op-een interactie met patiënten ook nuttig voor toekomstige vaccinatierondes, andersoortige preventie, bevolkingsonderzoek of gezondheidsvoorlichting. Artsen gaven echter aan hieraan vanwege hoge werkdruk geen structurele bijdrage te kunnen leveren. Alle respondenten zeiden duurzame oplossingen wenselijk te vinden. Het initiatief hiervoor moet wat betreft het merendeel bij de preventiesector liggen.

## Conclusie en aanbevelingen

De marktvaccinatieaanpak stoelt op het equity-principe. Niet door gelijke behandeling, maar juist door ongelijke behandeling op basis van behoefte werden barrières voor vaccinatie opgeheven. Deze aanpak, waarin laagdrempelige een-op-eencommunicatie tussen burgers en medische professionals in de wijk centraal staan, lijkt een belangrijke stap in het verhogen van de vaccinatiegraad in kwetsbare wijken.

In Rotterdam bleken de vrijwillige inzet en intrinsieke motivatie van medische professionals een randvoorwaarde voor succes. Aan zo’n belangeloze inzet zit echter een grens. Wij pleiten ervoor om dergelijke spontaan ontstane samenwerkingsnetwerken in de toekomst beter te faciliteren. Hiervoor is een stabiele samenwerkingsstructuur nodig die de inzet van medische professionals van diverse beroepsgroepen op de lange termijn mogelijk maakt en ruimte geeft aan pragmatische oplossingen van gezondheidsproblemen op locatie. Hiervoor zijn ruimere investeringen in de publieke preventieve gezondheidszorg noodzakelijk, zodat ruim baan kan worden gegeven aan nieuwe netwerken van professionals uit de zorgketen. Medewerkers van de GGD onderschrijven de gedachte dat de samenwerking met medische professionals in de wijk belangrijk is en moet worden gefaciliteerd en gestimuleerd. Om dit te bewerkstelligen zijn afspraken tussen GGD, RIVM, het ministerie van VWS en medische professionals van essentieel belang. Een gebrek aan samenwerking of wederzijds vertrouwen kan de opzet van dergelijke fijnmazige interventies, die naast grootschalige campagnes noodzakelijk blijken in het bereiken van bepaalde doelgroepen, in de weg staan.

Wanneer dergelijke samenwerkingsverbanden, waarin ook patiënten betrokken zijn, kunnen worden gefaciliteerd, blijft de intrinsieke motivatie van uitvoerend medisch personeel uiteraard onontbeerlijk voor succesvolle fijnmazige interventies. Onze ervaring is dat medische professionals ook klaarstaan om vergelijkbare preventieve uitdagingen aan te pakken, zoals het verhogen van de HPV-vaccinatiegraad, bevorderen van deelname aan bevolkingsonderzoeken en het keren van de stille epidemieën obesitas en diabetes. De COVID-19-vaccinaties op de Rotterdamse markten vormen het bewijs van de grote bereidheid tot samenwerking en creativiteit van medische professionals om volksgezondheidsproblemen aan te pakken en gelijke uitkomsten te realiseren.

## References

[1] Rotterdam-Rijnmond GGD. Dashboard COVID-19. https://gezondheidinkaart.nl/dashboard/dashboard/covid-19. Geraadpleegd op 23 mei 2022.

[2] Vader S, Uiters E, van der Lucht F, Smits C, Kroese F, de Bruin M (2021). Vaccinatiebereidheid en opleidingsniveau. Tsg Tijdschr Gezondheidswet.

[3] Cockerham WC, Hamby BW, Oates GR (2017). The social determinants of chronic disease. Am J Prev Med.

[4] Haybar H, Kazemnia K, Rahim F. Underlying chronic disease and COVID‑19 infection: a state-of-the-art review. Jundishapur J Chronic Dis Care. 2020. In press.

[5] Kamal A, Hodson A, Pearce JM (2021). A rapid systematic review of factors influencing COVID-19 vaccination uptake in minority ethnic groups in the UK. Vaccines.

[6] Willis DE, Andersen JA, Bryant-Moore K, Selig JP, Long CR, Felix HC (2021). COVID-19 vaccine hesitancy: race/ethnicity, trust, and fear. Clin Transl Sci.

[7] Deci EL, Ryan RM, van Lange PAM, Kruglanski AW, Higgins ET (2012). Self-determination theory. Handbook of theories of social psychology.

[8] Gaglio B, Shoup JA, Glasgow RE (2013). The RE-AIM framework: a systematic review of use over time. Am J Public Health.

